# High-Resolution Wind Tunnel Dataset of Gas Sensor Responses to Vapor Plumes in Scale Model Landscapes

**DOI:** 10.1038/s41597-026-06927-8

**Published:** 2026-04-03

**Authors:** Patrick Hinsen, Thomas Wiedemann, Dmitriy Shutin, Achim J. Lilienthal

**Affiliations:** 1 Institute of Communications and Navigation, German Aerospace Center (DLR), Oberpfaffenhofen, Germany; 2 Chair of Perception for Intelligent Systems, School of Computation, Information and Technology (CIT), Technical University of Munich (TUM), Munich, Germany

**Keywords:** Environmental monitoring, Electrical and electronic engineering, Information technology

## Abstract

Monitoring airborne pollutant emissions and discovering their sources is an important task that mobile robots and Unmanned Aerial Vehicles (UAVs) are well suited for. However, it requires suitable gas sensors compatible with these platforms, alongside sampling strategies that direct them to optimal sampling locations. This article presents a dataset comprising numerous wind tunnel experiments where a synthetic gas plume was systematically scanned using various gas and environmental sensors in a dense 3D grid pattern, complemented by additional sampling trajectories. The dataset allows direct comparison between low-cost metal oxide semiconductor gas sensors (MiCS-5524, MiCS-6814) and advanced photoionization detectors (PID-AH2), examining both their static and dynamic responses to the plume. Additionally, a scale model industrial facility enables the evaluation of gas dispersion models in complex settings under controlled wind conditions. The data support the comparison and evaluation of novel sampling strategies for mobile robotic sensor systems for gas distribution mapping and source localization, providing more realistic experimental data compared to computational fluid dynamics simulations. Meeting the need for objective comparability of approaches in robotic gas sensing, this experimental dataset can serve as a standard corpus and benchmark.

## Background & Summary

Detection, measurement, and modeling of airborne material, such as gases, particles, or aerosols, released into the atmosphere is known to be notoriously difficult. Their dynamics are known to follow complex, chaotic patterns due to time-varying advection effects, diffusion and the geometry of the surrounding environment. Yet in a number of situations it is crucial to be able to assess a spatial distribution of the matter of interest, identify potential sources or the amount of released material. Possible applications extend from response to man-made or natural disasters^1,2^, monitoring and assessing leakage rates from industrial complexes of facilities^3–5^, environmental monitoring^6^ and planetary exploration^7,8^, to name only a few.

The key challenge that is common to all aforementioned use cases are the complex spatio-temporal dynamics of the airborne material. While robots can collect large amounts of data using visual sensors, such as cameras, delivering millions of pixels per measurement, olfactory sensors typically employed on robotic platforms can well be compared to single-pixel cameras: chemical sensors output a significantly reduced amount of information as compared to their vision-based counterparts. In other words, one is forced to operate in a data scarce regime, while at the same time dealing with a highly dynamic spatial process. Moreover, sensing aspects play a significant role in data collection as well. Indeed, for *in-situ* sensors, one also needs to take into account the way a gas interacts with the gas sensor itself, or more specifically, how well a sensor is able to detect and quantify a substance in the air. Note that the *in-situ* sensor itself will also have an effect on the dispersion behavior of the gas, by the mere fact of being an obstacle to the gas flow. The effect is especially pronounced when considering gas sensors carried by flying platforms, such as unmanned aerial vehicles (UAVs). For example, the downwash effect produced by a quadcopter drone will cause a significant wake turbulence underneath, thus directly interacting with the quantities it is designed to measure in the first place.

One possible way to deal with these challenges is to improve sensing technologies (see e.g.,^9,10^). This remains an active research area in modern robot olfaction. Another approach, to which this paper in parts contributes, is to address this problem from two different, yet highly intertwined perspectives: (I) improve modeling of gas propagation, both experimentally and with simulation approaches, as well as (II) design more intelligent data collection approaches. The latter builds upon state-of-the-art, yet compact and affordable sensors, increased spatial sampling rate by using multiple robotic platforms, and finally, intelligent algorithms for selecting optimal sampling locations. In what follows, we will focus our attention specifically on several aspects related to this approach: experimental studies of gas propagation performed in controlled conditions and corresponding sensing aspects.

Historically, gas modeling is deeply rooted in computational fluid dynamics (CFD) methods. Although they are based on physical laws, and thus provide a firm foundation for their validity, these methods turn out to be extremely computationally intensive even for forward simulations. Inverse problems involving CFD, e.g., to identify source locations, or source release rates, turn out to be even more computationally challenging. On the other hand, an already trained data-driven model could be of significantly lower complexity. Their widespread use remains limited, however, mainly due to insufficient real-world data availability for training. This has a negative effect not only on training such models, but also on validating modeling and estimation techniques. Furthermore, taking into account the sensing aspects, such as the types of sensors used, calibration issues, and robot-plume interaction, further hinders developments of data-driven techniques.

**Fig. 1:**
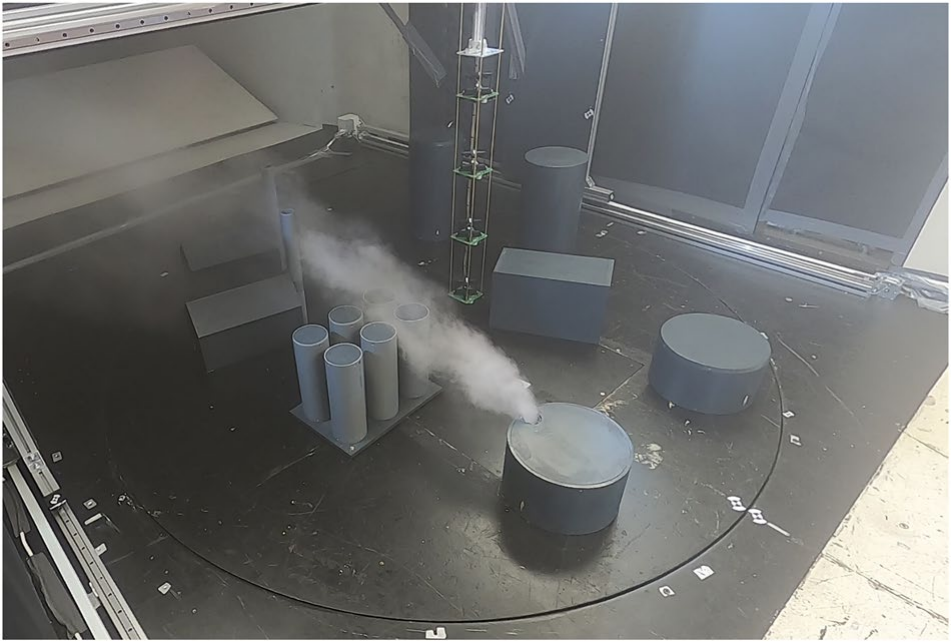
Setup of experiments in the wind tunnel for recording the Red:Vapor dataset.

Therefore, at the German Aerospace Center (DLR), we conducted a series of experiments to collect gas propagation data in 3D under controlled conditions. To this end, we have designed a sampling setup to take measurements of a steady-state gas/vapor plume inside a wind tunnel (see Fig. 1). This allowed us to create a 3D snapshot of the spatial gas distribution as seen by an *in-situ* sensor over an extended time period. In this snapshot, we could capture the concentration in high spatial resolution. Because our setup creates various flow regimes (laminar regions, disturbed flow and turbulence), the influence of these effects can also be studied. We designed a CNC sampling device that could place not only a single sensor, but multiple sensor packages arbitrarily in 3D in the gas plume, while also avoiding obstacles. Using this device, data from multiple experiments were recorded, which, to the best of our knowledge, are unique not only from the available resolution, but also with respect to the complexity of the scenarios.

The described measurements have been conducted in March 2023 in the Low-Speed Wind Tunnel (LST) facility operated by German-Dutch Wind Tunnels (DNW), jointly operated by the German Aerospace Center (DLR) and the Dutch Aerospace Center (NLR).

The dataset consists of a total of 39 measurements, recorded over four days: 8 rasterized sampling experiments, systematically recording the wind tunnel volume, lasting roughly 2 h each; 22 “fly-through” experiments, where the sensor probe behaves like a moving, e.g. UAV-mounted, sensor; and 9 purging runs of the wind tunnel, which allow us to study the drift of the sensors in the absence of gas. The resulting dataset we call Red:Vapor – *“Rasterized Experimental Data: Vapor Advection Plumes for Open Research”*.

Let us point out that although the collected data is unique in many respects, there are further datasets available that were collected specifically for gas distribution mapping and source localization using sensor networks and mobile robots. In^11^, the authors presented an early contribution by developing a real-world dataset for gas distribution and source localization with mobile robots. Despite low spatial resolution, the dataset was collected autonomously in 2D using a mobile platform. It was aimed at facilitating algorithm benchmarking and comparative studies, laying the groundwork for future research in mobile olfaction systems. While realistic, the control of external factors such as wind was significantly limited. Another 2D dataset has been published by the authors of^12^. In this wind tunnel setup, a 2D gas concentration distribution was recorded by simultaneously sampling a sensor array of 9 nodes, each containing 8 metal-oxide semiconductor (MOS) gas sensors. By repeating experiments, the authors recorded a 2D grid of 54 total sampling locations. While this dataset is similar in kind, it is fairly coarse in resolution when compared to the one produced in this work, and only 2-dimensional. The authors also restricted their experiments to the sensing principle of metal-oxide sensors. In^13^, another study was performed, now in 3D, using a grid of MOS gas sensors. The authors examined gas dispersion under both natural and artificially induced airflow conditions in an office room, providing empirical insights into the influence of airflow on gas concentration fields in three-dimensional spaces. The experiment and dataset was later extended^14^ by focusing on 3D gas distribution mapping and source localization aspects, emphasizing practical deployment scenarios. The latter studies highlighted a time-varying gas distribution pattern as well as a need for 3D gas sensing abilities for practical applications. These datasets are primarily experimental. Recently, a new study was released where generation of datasets was done using numerical CFD^15^. This CFD-based dataset was designed specifically for mobile robotic olfaction and provides synthetic but realistic gas dispersion scenarios that serve as benchmarks for testing algorithms. In contrast to that, our dataset offers both temporal as well as highly resolved spatial physical sampling in 3D of gas concentrations and accompanying environmental parameters, such as wind, temperature and humidity.

Some preliminary work has already been performed using the dataset presented here. In^16^, the authors performed identification of physical parameters of the gas dispersion models and characterized the temporal sensor response.

In the remainder of this article, we will describe the data collection setup, experiments, design decisions and introduce our data preprocessing routine throughout the Methods chapter. The structure of the data records is then described in the Data Records chapter. In the Technical Validation chapter, we discuss the technical validation of the collected measurements. This is followed by details on how to access data and code in the Code Availability and Data Availability chapters, dataset along with the accompanying source code for preprocessing and visualization.

## Methods

**Fig. 2:**
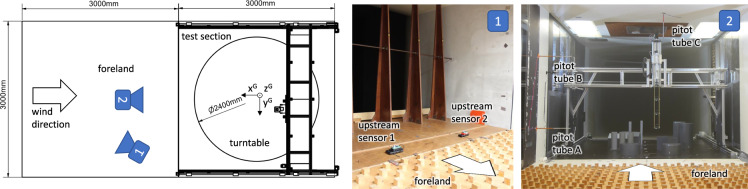
The schematic and pictures show the setup for the experiments in the wind tunnel. The white arrow indicates the wind flow direction.

In this section, we first introduce the wind tunnel in which the experiments took place. We then describe our sampling apparatus, the synthetic gas source and the sensors we used in detail. Additionally, we describe further auxiliary measurement equipment deployed in the tunnel. Toward the end of this section, we discuss the scale model setups we chose to place in the test section of the tunnel. Then, we explain the pattern in which we sampled the volume as well as our considerations with regard to timing. Lastly, we detail the preprocessing steps we performed to arrive at the published dataset. For an initial overview, we refer to Fig. 2.

Notation: We use the notation ${{\boldsymbol{p}}}^{A}={[{x}^{A},{y}^{A},{z}^{A},1]}^{T}$ to indicate a point in 3D space based on the coordinate frame *A* in our homogeneous coordinate system. A matrix ${{\boldsymbol{T}}}_{A}^{B}$ transforms a point from coordinate frame *A* into frame *B*, e.g. ${{\boldsymbol{p}}}^{B}={{\boldsymbol{T}}}_{A}^{B\,}{{\boldsymbol{p}}}^{A}$.

### Wind Tunnel Setup

The experiments were conducted in the Low-Speed Tunnel (LST) of DNW in the Netherlands. The LST provides a 3.0 m × 2.25 m stream cross-section with wind speeds between $0\,\frac{{\rm{m}}}{{\rm{s}}}$ and $80\,\frac{{\rm{m}}}{{\rm{s}}}$. For our experiments, we stayed near the low end of the wind speed range, utilizing speeds not above $10\,\frac{{\rm{m}}}{{\rm{s}}}$. The usable test section is 3 m long. The LST is a closed-circuit wind tunnel with the capability to ventilate the volume when required. In fact, in our chosen mode of operation, a fraction of the airstream was continuously replaced with fresh outside air.

The inflow airstream profile into the test section can be shaped by choosing a foreland in the upstream region of the tunnel. For our experiments, an “urban” foreland profile was installed (cf. Fig. 2), simulating an atmospheric boundary layer that one would expect over land. Its velocity *v* over height *z* can be approximated by

1 $$\frac{v(z)}{v({z}_{c})}={\left(\frac{z}{{z}_{c}}\right)}^{0.245}$$

with some reference height *z*_*c*_, according to DNW.

Embedded into the test section floor is a turntable of 2.4 m diameter. In this circular area, we installed our model setup. By turning the table and the model, we could emulate different wind directions.

The wind speed in the tunnel was monitored by three pitot tubes for operating the tunnel and regulating a constant wind velocity in the test section. The pitot tubes were installed along the hull of the tunnel, in the foreland area, just upstream of the test section. Pitot tube C was installed in the middle of the tunnel just 0.5 m below the ceiling. Pitot tubes A and B were mounted on the wall of the tunnel (approx. 0.4 m distance from the wall) at heights 1.5 m and 0.5 m. Technical drawings of this are provided along with the dataset. While only pitot tube C was used for control of the fan of the tunnel, all three pitot tubes were recording data. This is part of the data provided by the wind tunnel operator, which we introduce in section Data Records.

### CNC Probing and Coordinate Frames

Our experiments aimed to record a 3D map of a gas plume in a scale model facility of our choice. To this end, we designed a traversal mechanism to move a probe carrying various *in-situ* sensors in the 3D volume of the wind tunnel. Its function was to place said probe at programmed points in space and sample for a defined amount of time before moving to the next point. In this way, we could scan the whole test section of the wind tunnel efficiently and in an automated fashion with a high spatial resolution. The design was constrained by two requirements: (1) The mechanical assembly shall have minimal disturbance on the airflow in the tunnel and not affect the gas dispersion process. (2) The device needs to be rigid and stiff enough to hold the probe still without shaking caused by the airflow.

**Fig. 3:**
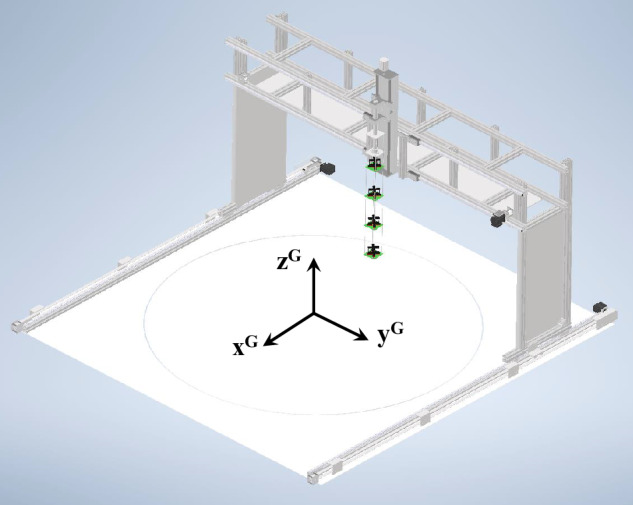
CNC machine for probing and the global coordinate frame.

To fulfill these requirements, we decided to design a traversal mechanism as a computer numerically controlled (CNC) gantry machine. The cross-section that was exposed to the airflow was reduced to a minimum and the sensors were held upstream of the device to reduce their effect on the measured gas dispersion. Its function can be compared to a CNC milling machine or a coordinate-measuring machine. It could move the probe along the three cartesian axes controlled by a computer program. Some renderings and pictures can be seen in Figs. 2 and 3.

Two linear rails were installed on the floor close to the walls of the test section, parallel to the airflow. Riding on these two rails, two vertical risers carried a gantry spanning across the wind tunnel volume. These risers and gantry had smooth inner walls and a slim profile and rose high enough above the scaled model such that the gantry’s influence on the airflow was minimal. On the gantry, two further linear rails allowed motion perpendicular to the airflow. On these rails rode another linear actuator, holding the probe and moving it up and down. This actuator was installed on the upwind side of the gantry, such that the probe was fully upwind of the gantry. For horizontal movement, cambelts driven by closed-loop stepper motors were pulling carriages running on the linear rails. Movement along the vertical axis was driven by a leadscrew mechanism. The whole assembly allowed for positioning the probe with subcentimeter accuracy. The computer control was realized by using a microcontroller board running the GRBL CNC firmware^17^. A single-board computer running Linux ran our command and control software that streamed motion commands as “G-Code” to GRBL, which handled the real-time control tasks and reported back instantaneous machine positions.

We will define multiple coordinate frames throughout this article. The global coordinate frame G is defined as the center of the turntable in the test section on floor level; see Fig. 3. The global X-axis *x*^G^ is horizontal and oriented into the airstream, such that coordinates increase when translating upwind and decrease when translating downwind. The Z-Axis *z*^G^ is vertical with coordinates increasing when translating upwards, and the Y-Axis *y*^G^ is chosen perpendicular to *x*^G^ and *z*^G^ following a right-handed coordinate system.

Transparent to the user, the CNC probing mechanism made use of the M coordinate frame. Its origin corresponded to the homing position of the machine, i.e. when all axes reach their limit switches. That is the minimal reachable *x*^G^ and *y*^G^ and maximal *z*^G^ location. This point was approached to initialize the machine as well as to square up the two X-axes relative to one another after a power cycle. The transformation between G and M, defined by ${{\boldsymbol{T}}}_{{\rm{M}}}^{{\rm{G}}}$, is a pure translation. It does not need to be explicitly calculated, as this transformation was performed by the CNC controller automatically by touch-probing the origin of G once after installing the machine. So, when commanding the machine to move to a new location, this was always done by giving coordinates in terms of G.

### Synthetic Gas Source

To generate a gas plume in our experiments, we need a synthetic source releasing gas or airborne substances in the tunnel. It was required that our sensor can detect the released substances. Besides, the source needed to be able to release gas continuously for several hours at a time, to allow for a dense sampling of the volume with sufficient sampling time per location. Additionally, we required a gas that is roughly neutrally buoyant, such that it did not rise to the ceiling or fall to the floor too fast. Lastly, the gas should be non-toxic to humans, non-combustible, and otherwise not hazardous or harmful to the environment.

**Fig. 4:**
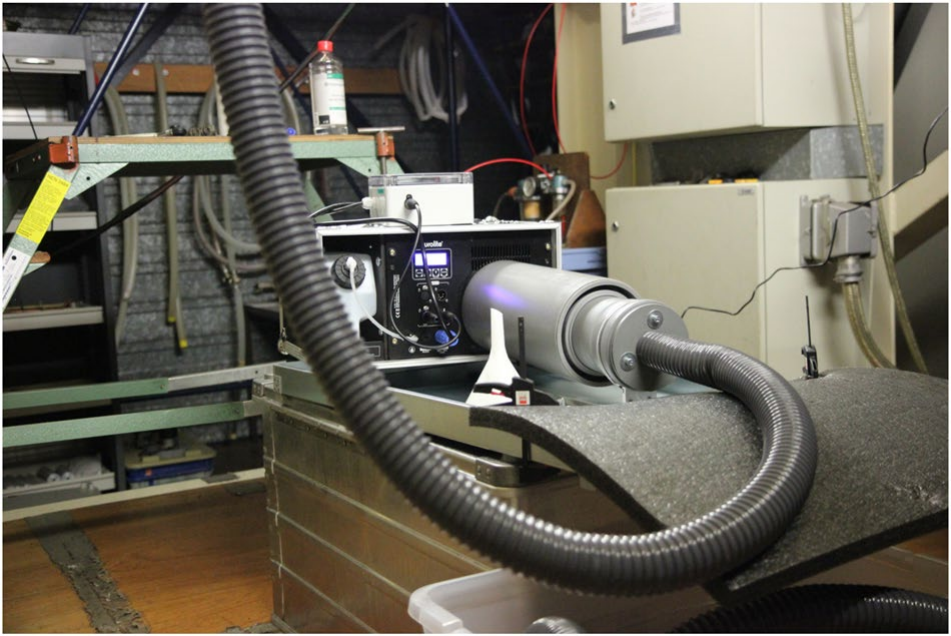
The fog machine was installed below the test section. A tube guided the fog to the outlet in the test section above.

We opted for an airborne vapor/aerosol plume that, despite being detectable by our sensors, can be captured visually. We installed a commercial fog machine *Eurolite N-130 Tour-Fogger* to function as our “gas” generator. This machine, with a heating power rating of 1300 W, works by evaporating a fog fluid to expel vapor/aerosol fog. Its pump can be regulated to achieve the desired level of volume flow. The emitted fog was then guided through a flexible hose into the designated outlet in the test section (see Fig. 4). In particular, for our setups, the fog was released through a hole in a cylindrical obstacle simulating a leakage in a gas tank (see Fig. 1). The position of the release varied throughout different experiment setups; however, it was on a fixed height of 0.18 m above ground.

We mixed a custom blend of fog fluid to achieve our desired properties. One batch of this mixture consisted of the following ingredients:

- 2 L propylene glycol (PG)
- 1 L triethylene glycol (TG)
- 1 L deionized water
- 20 mL or 40 mL ethanol

In some experiments 20 mL of ethanol were used, in some 40 mL. Compared to common commercially available fog fluids, this mixture has a reduced concentration of water, resulting in an overall lower enthalpy of evaporation. This is favorable when running the machine continuously. The triethylene glycol makes the plume more visible in photos and video recordings. The propylene glycol is added as the main constituent; we also found that it is being detected by some of our sensors (cf. Sec. Preprocessing). The ethanol is added as the main tracing substance, as many sensors are highly sensitive to ethanol even in low concentrations. It is noteworthy that the fluid left the orifice of the fog machine at a very high temperature (above the boiling point of water). However, it was immediately mixed with ambient air through a venturi at the orifice to increase the volume flow while reducing the release velocity. When guided through a tube to the outlet in the test section, the fog was further cooled down. One point of caution that should be mentioned at this point is that the released fog is a mixture of gas and aerosol. Care needs to be taken when comparing it’s transport behavior to pure gasses. Aerosol droplets and their condensation also introduce slight nonidealities, as the primary gas plume and local evaporation of droplets combine in the sensor signals.

### Sensors and Sensor Layers

Our choice of sensors for the experimental campaign was primarily motivated by the constituent chemicals in the fog fluid. Furthermore, we aimed to compare low-cost sensors, suitable for consumer devices and swarm systems, to more sophisticated, expensive sensors.

As low-cost sensors, we chose several Metal Oxide Semiconductor (MOS) sensors. Specifically, we integrated the MiCS-5524 of SGX Sensortech, which, according to the datasheet, shows a good sensitivity to ethanol^18^. Further, we made use of the OX and NH3 channels of the MiCS-6814^19^, which are additionally sensitive to NO_2_, C_3_H_8_, and C_4_H_10_. As is common for MOS sensors, these sensors are cross-sensitive to other substances. During our trials, we observed sensitivity also primarily to the propylene glycol (PG) component of the fog fluid. While some MOS sensors can respond rapidly to concentration changes, their response is nonlinear, and may be affected by hysteresis and be rather noisy.

Besides these two MOS sensors, we integrated an Alphasense PID-AH2 photoionization detector (PID) to serve as a “gold standard” for comparison. PID sensors have fast response and recovery times, show less aging and drift, as well as a linear relation between voltage output and observed concentration values. This allows us to compare low-cost MOS sensors to PID sensors, in terms of suitability and measurement quality.

Lastly, a selection of environmental parameters was recorded co-located with the gas sensors. For pressure, relative humidity, and temperature (PHT), we installed Bosch BME680 environmental sensors. To obtain a 3D wind vector, we chose Anemoment TriSonica Mini 3D ultrasonic anemometers. As an auxiliary measurement, the PHT readings from the anemometer’s internal sensor were also recorded.

**Fig. 5:**
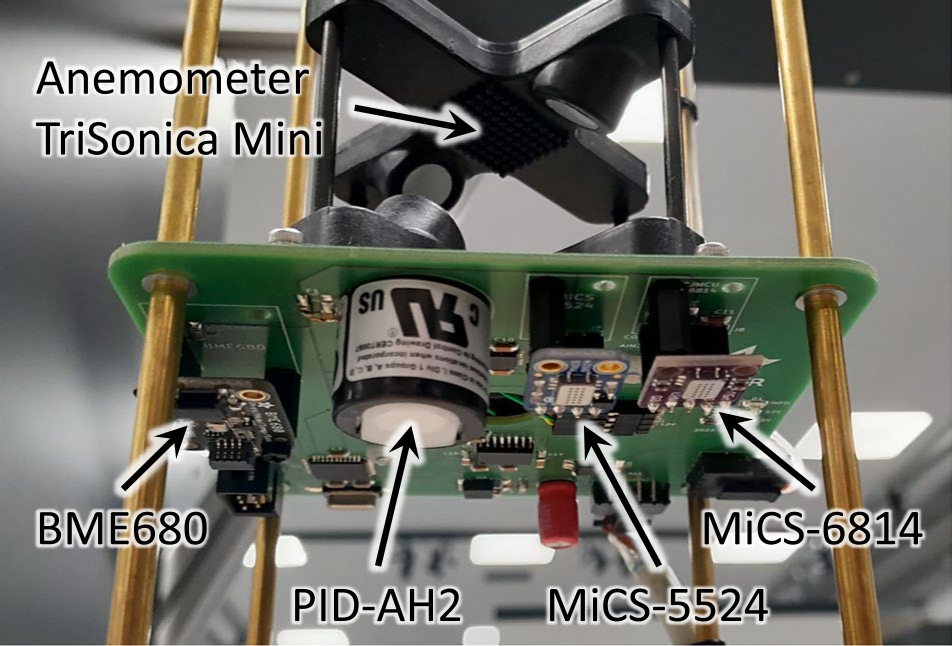
Photo of one individual sensor platform. The different gas and PHT sensors were mounted on the underside. The ultrasonic anemometer was installed on top.

**Fig. 6:**
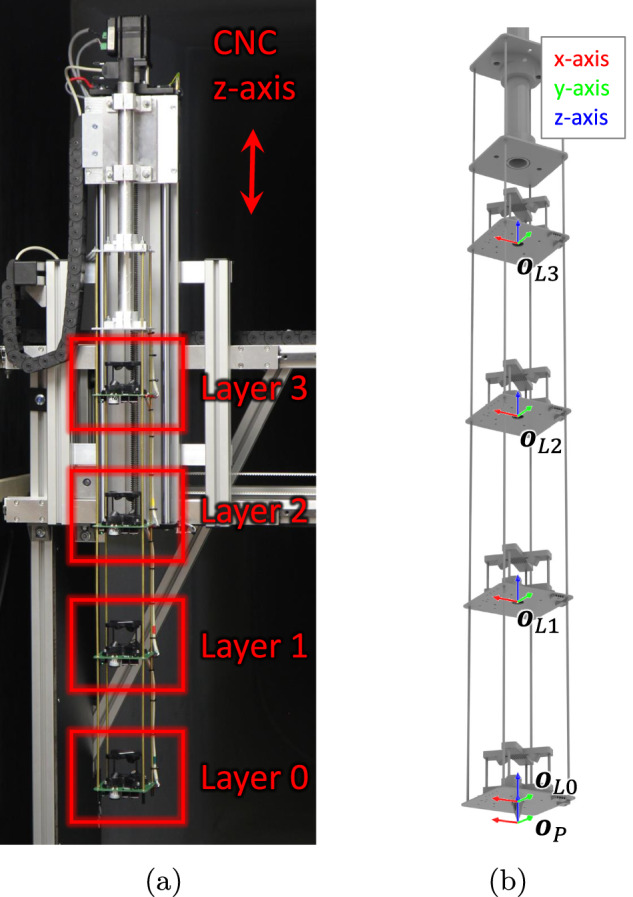
Sensor probe consisting of four equal sensor layers; in (b), the different coordinate frames for different layers are depicted.

To facilitate efficient scanning of the wind tunnel volume, we designed a sensor probe with four identical layers (cf. Fig. 5), referred to as sensor platforms or layers. In this way, we could sample at four different points in space at the same time and quarter the time required for our experiments compared to a single sensor layer. The layers were equally spaced every 24 cm in the vertical axis (cf. Fig. 6). As spacers between the layers, we used four 4 mm brass tubes. The stack of layers was still rigid with minimal vibration and oscillation when operating in the tunnel. The slender tubes reduced the disturbance from the probe on the airflow in the tunnel. By translating the array of four layers with our CNC probing mechanism to only 3 levels in the Z-axis in increments of 8 cm, we achieved a raster of 12 equally spaced vertical samples at 0 cm, 8 cm, 16 cm, 24 cm, … 88 cm.

Each sensor platform consisted of a 10 cm × 10 cm printed circuit board (PCB), on which the sensors were mounted. The analog signals from all gas sensors were digitized using an ADS112C04 16-bit Sigma-Delta Analog Digital Converter (ADC) and read using an Atmel AVR ATmega324 microcontroller. The digital sensors, i.e., BME680 and the anemometer, were attached directly to the AVR. This configuration was replicated on each layer, and they were connected via a bus providing power and an RS-485 data line. The exact circuit schematic is provided as part of the dataset.

We operated each of the sensors as suggested by the manufacturers’ recommended application circuits. For the PID sensors, this is a constant voltage supply in conjunction with the internal voltage regulator. For the MOS sensors, this consists of a series resistor between heating resistance and constant voltage supply to regulate the temperature of the sensitive layer, and a series load resistor forming a voltage divider with the sensor resistance. We want to stress that these circuits are a solid baseline choice and should facilitate comparability to other works, but are not representative of the leading edge in sensor system research. To highlight just one field, there exist a substantial body of work in regards to MOS sensor temperature control and modulation.

The RS-485 data line was driven by the single-board computer, which also interfaced with the CNC machine controller, as previously mentioned. This computer generated a 10 Hz clock and issued a capture signal as RS-485 broadcast over the data line at that frequency. This ensured that sample capture is initiated on all layers synchronously to at least the *μ*s. Capture of all channels is then completed within approx. 2 ms, which is  ≪ 100 ms, i.e. fast in relation to our sample rate, which in turn is fast in relation to the gas propagation dynamics. Data was read out over the bus line, and combined with the live positioning feedback stream from the CNC controller (as x_recorded, y_recorded, z_recorded). This data was then stored in an InfluxDB v2 time series database, also running on the single-board computer. Each sample was indexed by the NTP timestamp at which the trigger signal was issued. Additionally, whenever the machine was not transitioning in between grid cells, the sample was tagged with a set of x,y,z coordinates. Tagging only began when the machine arrived at a standstill. That means that samples tagged with x,y,z coordinates have positively been recorded in the designated location. This was ensured by flushing the CNC machine’s motion buffer, synchronizing the reported and actual position.

Recall that we defined G as being the global coordinate frame with its origin in the center point of the turntable. In the following, we introduce additional coordinate frames for the sensor and the sensor layers with the help of Fig. 6. The actual values and transformation matrices are given in Table 1. The origin ${{\boldsymbol{o}}}_{{\rm{P}}}^{{\rm{G}}}$ of the sensor probe (i.e., stack of layers) is the tip of a touching probe mounted 2.5 cm below layer 0. In the dataset, the columns x_recorded, y_recorded, z_recorded correspond to the momentary location of the probe’s origin as reported by the CNC controller, i.e. ${{\boldsymbol{o}}}_{p}^{{\rm{G}}}={\left({x}^{G},{y}^{G},{z}^{G},1\right)}^{{\rm{T}}}$ (homogeneous coordinates). The transformation of a location in the probe’s frame to the global coordinate frame is given by ${{\boldsymbol{T}}}_{{\rm{P}}}^{{\rm{G}}}$. The origin of the layer frames ${{\boldsymbol{o}}}_{LX}^{{\rm{P}}}$ is the center of the PCB *X* = 0, …, 3. ${{\boldsymbol{T}}}_{{\rm{LX}}}^{{\rm{P}}}$ transforms locations on the PCB to the probe frame. In the dataset, the columns x, y, z correspond to the nominal location of each *layer* in global coordinates, i.e. ${{\boldsymbol{o}}}_{LX}^{{\rm{G}}}$. These values might be empty or “nan” as long as the CNC machine has not reached these commanded locations, or no nominal location has been sent to the CNC machine, yet (e.g., at the beginning of an experiment).

**Table 1:** Coordinate Frames. Units are always meters.

Symbol	Values	Comments
${{\boldsymbol{o}}}_{p}^{{\rm{G}}}$	$${\left[\begin{array}{cccc}{x}_{P}^{G} & {y}_{P}^{G} & {z}_{P}^{G} & 1\end{array}\right]}^{T}$$	location of the probe’s origin (touch tip) in global coordinates
${{\boldsymbol{T}}}_{{\rm{P}}}^{{\rm{G}}}$	$$\left[\begin{array}{cccc}1 & 0 & 0 & {x}_{P}^{G}\\ 0 & 1 & 0 & {y}_{P}^{G}\\ 0 & 0 & 1 & {z}_{P}^{G}\\ 0 & 0 & 0 & 1\end{array}\right]$$	transformation from probe’s coordinate frame to global coordinates
${{\boldsymbol{T}}}_{{\rm{L0}}}^{{\rm{P}}}$	$$\left[\begin{array}{cccc}1 & 0 & 0 & 0\\ 0 & 1 & 0 & 0\\ 0 & 0 & 1 & 0.025\\ 0 & 0 & 0 & 1\end{array}\right]$$	transformation from Layer 0 frame to probe coordinates
${{\boldsymbol{T}}}_{{\rm{L1}}}^{{\rm{P}}}$	$$\left[\begin{array}{cccc}1 & 0 & 0 & 0\\ 0 & 1 & 0 & 0\\ 0 & 0 & 1 & 0.265\\ 0 & 0 & 0 & 1\end{array}\right]$$	transformation from Layer 1 frame to probe coordinates
${{\boldsymbol{T}}}_{{\rm{L2}}}^{{\rm{P}}}$	$$\left[\begin{array}{cccc}1 & 0 & 0 & 0\\ 0 & 1 & 0 & 0\\ 0 & 0 & 1 & 0.505\\ 0 & 0 & 0 & 1\end{array}\right]$$	transformation from Layer 2 frame to probe coordinates
${{\boldsymbol{T}}}_{{\rm{L3}}}^{{\rm{P}}}$	$$\left[\begin{array}{cccc}1 & 0 & 0 & 0\\ 0 & 1 & 0 & 0\\ 0 & 0 & 1 & 0.745\\ 0 & 0 & 0 & 1\end{array}\right]$$	transformation from Layer 3 frame to probe coordinates
${p}_{{\rm{MiCS}}-5524}^{{\rm{LX}}}$	$${\left[\begin{array}{cccc}0.04 & 0.009 & -0.01 & 1\end{array}\right]}^{T}$$	location of the MiCS-5524 gas sensor on the sensor layer X
${p}_{{\rm{MiCS}}-6814}^{{\rm{LX}}}$	$${\left[\begin{array}{cccc}0.04 & 0.026 & -0.01 & 1\end{array}\right]}^{T}$$	location of the MiCS-6814 gas sensor on the sensor layer X
${p}_{{\rm{PID}}-{\rm{AH2}}}^{{\rm{LX}}}$	$${\left[\begin{array}{cccc}0.037 & -0.012 & -0.017 & 1\end{array}\right]}^{T}$$	location of the PID-AH2 gas sensor on the sensor layer X
${p}_{{\rm{BME}}680}^{{\rm{LX}}}$	$${\left[\begin{array}{cccc}0.023 & -0.038 & -0.01 & 1\end{array}\right]}^{T}$$	location of the BME680 environment sensor on the sensor layer X
${p}_{{\rm{TriSonica}}}^{{\rm{LX}}}$	$${\left[\begin{array}{cccc}0 & 0 & 0.03 & 1\end{array}\right]}^{T}$$	location of the center of the TriSonica anemometer on the sensor layer X
${p}_{{\rm{up1}}}^{{\rm{G}}}$	$${\left[\begin{array}{cccc}7.5 & 0.5 & 0 & 1\end{array}\right]}^{T}$$	location of upstream sensor 1 in the tunnel (global coordinate frame)
${p}_{{\rm{up}}2}^{{\rm{G}}}$	$${\left[\begin{array}{cccc}7.5 & -0.5 & 0 & 1\end{array}\right]}^{T}$$	location of upstream sensor 2 in the tunnel (global coordinate frame)

The location of the gas and environment sensor on the PCB is also summarized in Table 1 with respect to the layer frame *LX*. As can be seen from the value, and the picture in Fig. 5, the sensors were mounted below the PCB.

As an exception, the anemometer was placed on top of the PCB. When compared to the datasheet, the anemometer was rotated by 45^∘^ to get the stringers of their superstructure aligned with the brass spacers between the sensor layers. This was done to minimize the influence of these stringers on the wind measurement. Despite the rotation, the values of the wind measurements reported in the dataset are already transformed back to the global coordinate system. Thus, wind-u, wind-v, wind-w correspond to the airflow velocity components in *x*^*G*^, *y*^*G*^, *z*^*G*^ directions.

Over the course of the experiments, we observed significant amounts of condensation of the fog fluid on the sensor probe, including on the sensors themselves. The lowest sensor layer was most affected by this, with decreasing volumes of fog impinging on the layers as height (*z*^*G*^) increases. To ensure suitable measurement quality, we opted to swap sensor units for wear leveling purposes, or replace them entirely, once the live readout of the measurement data throughout the experiment showed signs of deterioration. Because this information may be important to users wanting to correlate PID/MOS sensitivities across experiments, we will detail the exact swaps/replacements below.

At the beginning of our measurements, we equipped all sensor circuits with fresh, sealed PID and MOS sensors.

We replaced the MOS sensors for a complete fresh set on 2023-03-08 at 08:26 UTC. Two wear leveling changes were performed w.r.t. the PID sensors on day 2025-03-09. Table 2 indicates how and when the sensors A-F were implanted and switched between the sensor layers. As can be seen there, we first removed PID specimen A, which started out on the lowest layer. In the second change, we removed PID specimen B, which started out on the second layer. Both A and B were swapped with one of the specimens from the upper layers, which experienced less condensation load. New PID specimens were emplaced there, with the rationale being that the low concentration values present at these heights would best be resolved by completely fresh PIDs.

**Table 2:** PID sensor swaps/replacements.

Layer	2023-03-07 9:00 – 2023-03-09 14:28	2023-03-09 14:28 – 2023-03-09 16:15	2023-03-09 16:15 – 2023-03-10 14:00
**3**	D	**E**	E
**2**	C	C	**F**
**1**	B	B	**C**
**0**	A	**D**	D

Times are in UTC. The table shows the individual PID sensor IDs A–F, which map to serial numbers.

The PID sensors on the upstream sensors were not swapped after the start of the experiments, as they showed no sign of deterioration.

### Auxiliary & Upstream Sensors

In addition to the sensors placed on the sensor probe, we placed some additional sensors in the upstream section of the wind tunnel. These are, first and foremost, the previously mentioned pitot tubes on the outer lining of the wind tunnel. These measured static and dynamic pressure and allow for accurate derivation of the onstream velocity of the air in the wind tunnel. To get out of the region of influence of the boundary layer, the pitot tubes were installed with an offset from the walls.

**Fig. 7:**
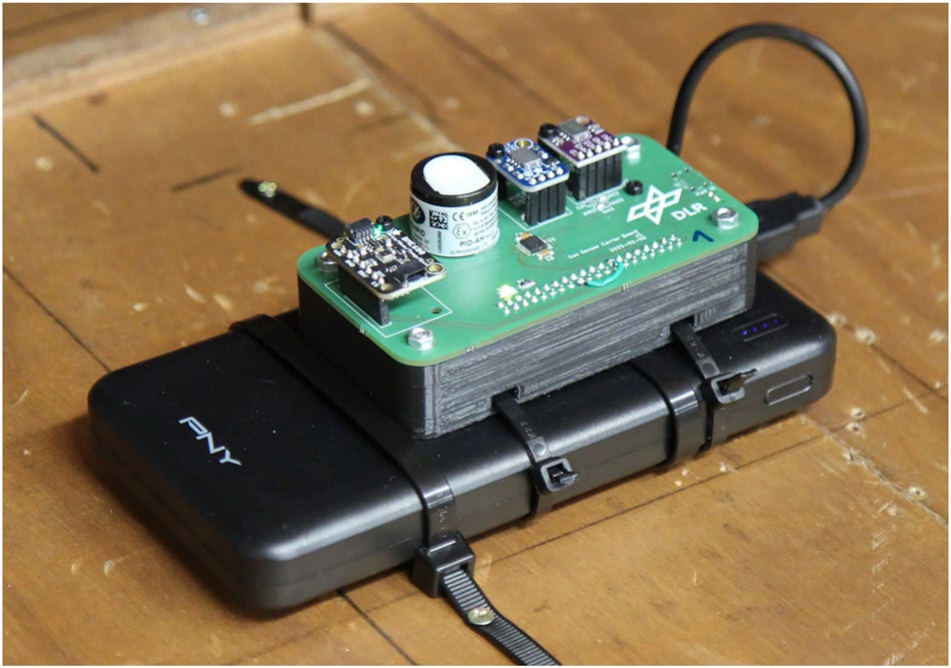
One of the upstream sensors for monitoring background concentration.

Aside from these wind measurements, we also placed two modified sensor platforms in the upstream regions of the wind tunnel on the ground, directly after the settling chamber and just before the foreland. They were located at ${{\boldsymbol{p}}}_{{\rm{up}}1}^{G}={[7.5,0.5,0,1]}^{T}$ and ${{\boldsymbol{p}}}_{{\rm{up}}2}^{G}={[7.5,-0.5,0,1]}^{T}$ (all units in meters). These upstream sensors were almost identical to the regular sensor platforms, aside from the fact that they lacked the TriSonica anemometers and were powered by power banks (see Fig. 7). Further, they streamed their recorded data via WiFi to the central database. Apart from that, they also made use of a MiCS-5524 and MiCS-6814 MOS sensor, the PID-AH2 sensor, and the BME680 for environmental parameters. The purpose of these sensors was to supply us with background measurements of the gas concentrations for all sensor types. This was necessary because the wind tunnel is a closed circuit. That means that over time, the vapor released into the tunnel accumulates, causing background concentrations to increase. Even though the tunnel was operated in a configuration where a part of the air is continuously replaced with fresh ambient air, we did observe an increase in sensor readings that cannot be neglected ($\le 1\,\frac{{\rm{ppm}}}{{\rm{h}}}$ measured on the PID channel; per-experiment max. amplitudes are in the range of 12.1 ppm to 41.8 ppm). That being said, the concentration signals are still very prominent above the level of the background (cf. Fig. 15 at the end of this paper). We used the upstream sensors to monitor the background concentration during our experiments. As such, they are part of the dataset. Furthermore, during the experiments, our decision on when to fully ventilate the tunnel volume was based on their reading. The measurements from these sensors are also used in our pre-processing steps to subtract the background concentrations in the tunnel from the individual sensor readings (cf. Sec. Preprocessing).

### Model Landscape

**Fig. 8:**
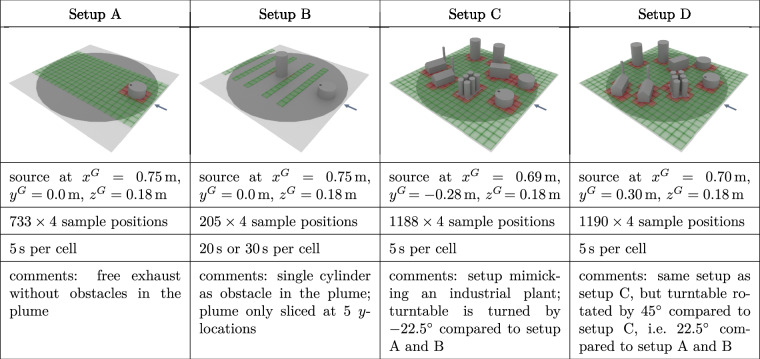
The table summarizes the four setups used in the experiments. The pictures depict the setup of the obstacles on the turn table, i.e. gray circle. The green grid shows all locations (center of the cells) where measurements have been taken at all heights. In red cells, obstacles needed to be avoided, and not all heights are measured. The positions stated here have been measured in the physical model. The sample counter includes the initialization routine. The arrow indicates the wind direction.

To study the effect of gas leakages and releases, we chose a model landscape mimicking an industrial facility. This model facility was composed of various buildings, tank farms, and smoke stacks at an approximate 1:50 scale. This model was installed on the turntable in the wind tunnel volume, allowing us to rotate it to emulate different wind directions. One of the buildings, a model of an oil/fuel storage tank, functioned in all experiments as the outlet of our synthetic gas source. Its position on the turntable remained unaltered throughout all experiments; it was, however, rotated with the turntable. We also reconfigured the model landscapes, and removed buildings to have an unobstructed gas dispersion as a reference or to study the gas interaction with a single building. In total, we used four different configurations of buildings. These four landscapes are summarized in Fig. 8. In setup A, we cleared all obstacles aside from the source itself to get a reference of the plume forming in the absence of obstacles, aside from the ground. Setup B consisted only of the source and a tall, cylindrical building. This was of particular interest because it is simple to model geometrically, and yet we expected interesting airflow and plume dispersion behavior due to a Karman vortex street forming in the wake of the building. Setup C and D were the most complex ones, with many more buildings placed. The setups were identical, aside from a rotation of the turntable of either 22.5° or −22.5°. All Model Landscapes are provided as 3D object files in the dataset.

Scaled-down models require some discussion of scaling effects in wind tunnel models, where times, distances and speeds are related through the dimensionless Reynolds number, among others. As with all scale models, scaling factor and wind speed were chosen as a compromise. We wanted our chosen models to present some variation in flow regimes (laminar and turbulent regions, vortex shedding). Our models are scaled down to also offer some variation in presented building geometries. To summarize the effects in brief, scaling up our models to a real-world scale would reduce wind speeds by the same factor, as dictated by the Reynolds number. On the other hand, the physical sensors were of course not subject to this; they are already “life-size” in the wind tunnel setup, including their time constants for response and recovery. Adjusting the wind speed upwards in the scale model would be detrimental to their ability to resolve oscillations, the frequency of which increases with the wind speed and the scaling factor, as dictated by the Strouhal number. Similarly, for the effects of the impinging airstream on the sensors (gas exchange with internal cavities, temperature exchange, …), we would preferably have kept the wind speed 1:1, as the wind was essentially affecting the sensors 1:1. Ultimately, we chose the wind speed such that the plume was allowed to expand to a reasonable diameter in relation to the landscape and the grid resolution; that is, high enough to serve advection, yet low enough to not prevent the plume from expanding. This means that while the transfer to full-scale is not fully ideal, the dataset does offer a wide variety in the discussed aspects.

### Sampling Patterns

With our experiments, we aimed to sample the 3D space around the model landscape where the plume is forming. We executed two categories of sampling experiments. The first we refer to as grid-based sampling, where the entire volume is systematically scanned. The second category we call fly-through experiments, in which the probe moves continuously along a predefined path. These experiments should simulate a flight of an Unmanned Aerial Vehicle (UAV) carrying the sensors to also capture the dynamic response when traversing the plume. Both types of experiments were repeated for low ($1\,\frac{{\rm{m}}}{{\rm{s}}}$ to $2\,\frac{{\rm{m}}}{{\rm{s}}}$) and high ($3\,\frac{{\rm{m}}}{{\rm{s}}}$ to $4\,\frac{{\rm{m}}}{{\rm{s}}}$) wind speeds. The corresponding mean wind speed for each experiment is provided in the metadata, and the overview table in the dataset. In the following subsections, we will explain the sampling patterns in more detail. While we aim to be rigorous in our explanation of how these patterns were designed and executed, we also kindly refer the reader to the exemplary videos included as part of the dataset for a visual introduction to these sampling patterns.

#### Grid-Based Sampling Patterns

To sample the 3D space around the model landscape systematically, for each model landscape setup, we computed a grid-based sampling pattern. In general, we divided the space into a regular grid, with cells of size 12 cm × 12 cm horizontally, 8 cm vertically, roughly aligning with the physical dimensions of the sensor layers. The goal of the sampling pattern was to have each cell visited at least once by a sensor layer. This grid resolution and the dimension of the test section resulted in a maximum grid dimension of 20 × 20 cells horizontally and 12 cells vertically, covering a volume of 240 cm × 240 cm × 96 cm. However, for certain experiments, we narrowed down the region of interest that is sampled. Furthermore, when moving the probe, we assumed a horizontal 14 cm × 14 cm safety box around the sensor layers. Whenever this safety box around one grid cell center would intersect with a building, we treated this as a collision and thus did not visit the respective cell. Note that, as can be seen from Fig. 8, there were no overhanging structures and each free grid cell could be approached safely from above.

Figure 8 shows the different setups with the obstacles together with the sampling grid projected onto the ground. All cells colored in green were free for all heights of the sensor probe, i.e. 4 × 3 samples could be collected (4 layers sampling at 3 different heights). Cells colored in red would have caused a collision with an obstacle at at least one height of the probe. Thus, the probe skipped measurements at some heights to avoid collisions.

It is noteworthy that for positions where we were forced to raise the probe to get clearance over an obstacle, we did not discard the measurements of the upper-most layers. Due to this fact, in these locations, we obtained additional samples at higher than usual altitudes, i.e. ***z***^*G*^ > 0.90 m. However, please keep in mind that at these heights the influence of the gantry might not have been negligible.

The sensor probe always started in the corner ${{\boldsymbol{p}}}^{{\rm{G}}}={(+1.140{\rm{m}},-1.140{\rm{m}},0.00{\rm{m}})}^{T}$, which we considered as clean air (upstream of the source). This location was also close to the two pitot tubes A and B, allowing cross-validation of the ultrasonic anemometer readings with the readings of the pitot tubes. At this special location, we sampled at 4 heights (${{\boldsymbol{z}}}_{P}^{{\rm{G}}}\in \{0.24{\rm{m}},0.16{\rm{m}},0.08{\rm{m}},0.00{\rm{m}}\}$). This created an overlap, where three locations are sampled by more than one sensor layer. The layer pairs L0&L1, L1&L2 and L2&L3 each sampled the same grid cell once. This allows users to compare, calibrate or cross-validate the sensors across layers, because we can assume that they were exposed to identical conditions.

**Fig. 9:**
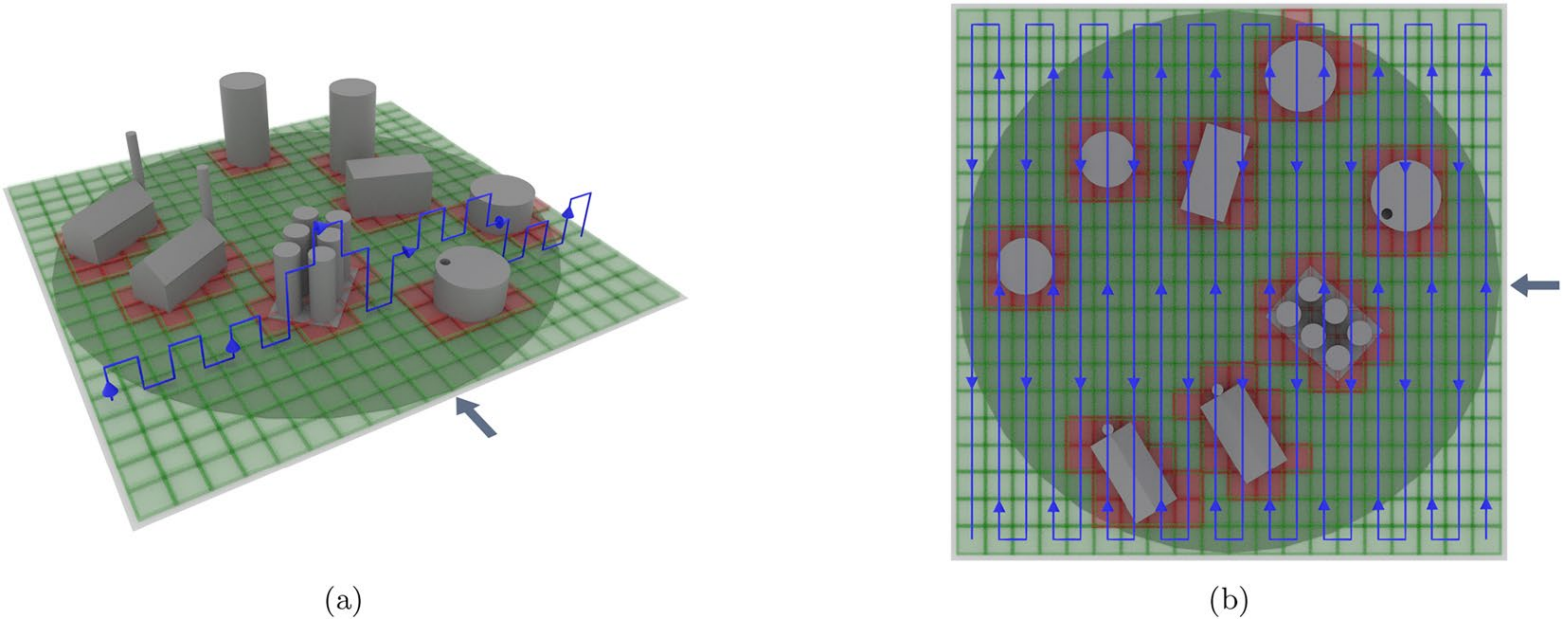
The two plots illustrate the sampling trajectory, marked in blue, of the probe for setup D. The gray arrow indicates the wind direction; wind blows along the *x*^*G*^-axis in negative direction. The probe scanned across grid cells first in *z*^*G*^ direction, then in *y*^*G*^ direction and lastly in *x*^*G*^ direction. The rendering in (**a**) shows an example of a single Y-Z row, and a top-down view of the trajectory is shown in (**b**).

The grid cells were scanned first in *z*^*G*^ direction, then in *y*^*G*^ direction, and lastly in *x*^*G*^ direction as illustrated in Fig. 9. When needing to avoid an obstacle, the probe moved up to a safe height above the obstacle to sample the cell. In the general case, if no obstacles were present, the probe sampled at 3 heights. There is one exception to this rule: After completing every other Y-Z sampling row, we sampled at 4 instead of 3 heights, similarly to the very first cell. Again, that means that there are redundant measurements for certain heights. This allows users of the dataset to cross-calibrate the sensors of adjacent layers periodically throughout the experiment. Especially MOS sensors are known to potentially drift over time.

In each cell, the probe stopped and remained stationary for a certain time (typically 5 s; 20 s or 30 s for setup B). This allowed for the transient response of the sensors to converge to the concentration in the cell. It is to be expected that 5 s are not long enough for the entire sensor step response to converge. This amount of time was chosen, however, as a compromise for setups C and D. It takes into account the total grid cell count and keeps the total experiment runtime to a reasonable length. In experiments with fewer grid cells (i.e., setups A and B), we were thus able to increase sampling time per cell. The exact sampling time is given in the dataset as metadata for every experiment.

Measurements were taken continuously, also during the movement of the probe, always labeled with the CNC machine’s position readout (x_reported, y_reported, z_reported), and timestamped. But only once the probe has positively come to a standstill, are the tags x, y, z applied; cf. Sec. Sensors and Sensor Layers.

#### Fly-Through Sampling Patterns

**Fig. 10:**
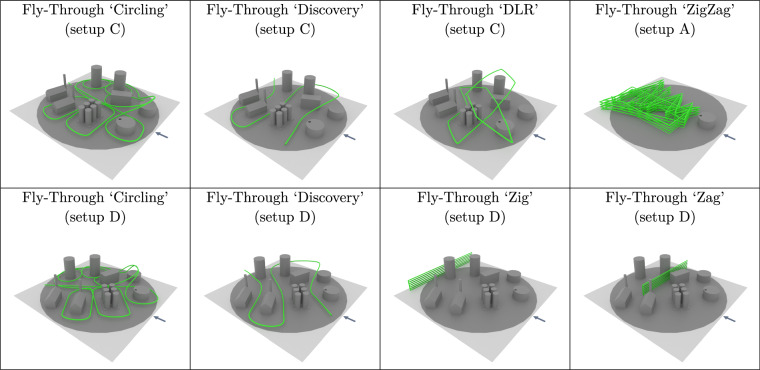
Trajectories of the different Fly-Through sampling experiments, shown as green lines.

Aside from the grid-based sampling pattern, we also employed sampling strategies that were not constrained to the described grid. These trajectories were modeled to mimic the flight of a UAV. In contrast to the grid-based sampling, the probe moved continuously without stopping and waiting. In other words, the probe “flew through” the model landscape. This data also captures the dynamic behavior of the sensors when entering or leaving the plume for the different sensor types. To this end, we designed the fly-through sampling patterns depicted in Fig. 10. The figure shows the trajectories of the probe, our naming of the pattern as used in the dataset (i.e., Circling, Discovery, DLR, Zig, Zag, ZigZag), and the setups used.

Note that, since the probe was not stopping at the commanded waypoints, the dataset only contains x_reported, y_reported, z_reported corresponding to the location of the probe’s origin ${{\boldsymbol{o}}}_{p}^{{\rm{G}}}$, but not the location of the individual layers. However, these locations can be easily calculated based on the coordinate transformation summarized in Table 1.

#### Purging Runs

Whenever time permitted, we ventilated the wind tunnel in between experiments by running the wind tunnel at a higher speed with the ventilation hatches fully opened. We refer to these procedures as “purging runs”. These periods constitute their own kind of “sampling pattern” – just without motion. The machine was commanded to position the sensor probe in the location closest to the pitot tubes A and B, the same one that was visited at the beginning of each experiment. From then on, the probe was held stationary and data were recorded continuously. The purpose of these experiments was to allow for analysis of the recovery period of the sensors, of their response to clean-air samples, and potentially of their drift behavior in clean air.

Note that the conditions were much less controlled during these experiments. The wind speed may have been adjusted even during a run, or even stopped entirely, after the tunnel has been purged sufficiently. The actual wind speed achieved can be recovered from the 7 wind speed sensors. Note that the higher wind speeds may have excessively chilled the MOS sensitive layers. While users should be aware of this fact, it is another effect that may be studied. During the runs, the side doors of the test section may have been opened, and personnel may have entered the wind tunnel test section to clean the machine from condensation.

### Preprocessing

**Fig. 11:**
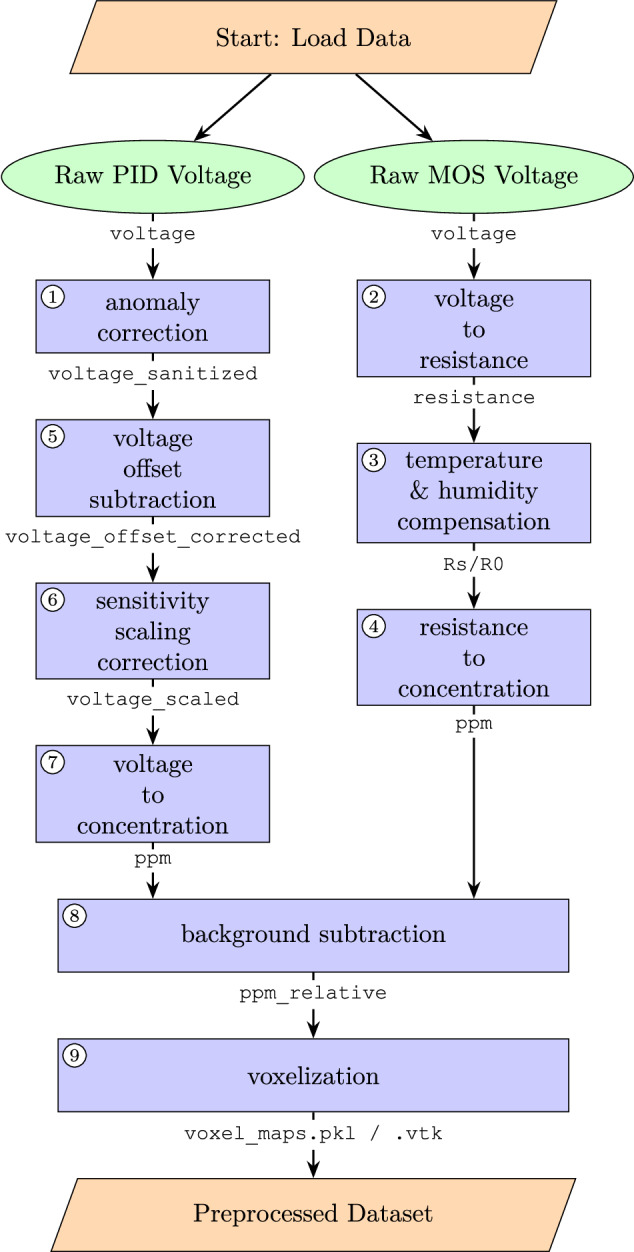
Preprocessing steps summarized as a flow chart. Labels written in monospace font indicate the dictionary keys or filenames that the quantities are available as. The numbers refer to the subsections that explain the respective steps.

All raw data samples are provided as-is and in full in the dataset. Users are free to perform their own preprocessing steps as desired. As a starting point, we additionally provide preprocessed versions of the dataset, along with the program code used for preprocessing. The code is openly available, as described in Sec. Code Availability. Here, we will describe the effects we compensate for and how it is done. These steps are motivated by the fact that we consider one of our main deliverables to be a 3D map of the background-subtracted gas concentrations in the wind tunnel section. For this, we need to be able to subtract the background concentration, compensate for humidity and temperature, etc. All preprocessing aims at a concentration-proportional signal, i.e., in ppm above background. The individual steps are explained in more detail in the remainder of this chapter, and summarized in the form of a flow chart in Fig. 11.

#### Step 1 – Anomaly Correction for Upstream Sensors

Unfortunately, we observed some minor anomalies in the recorded data that we need to correct. Unlike the sensor platforms, which regulate their power using low-dropout (LDO) linear regulators, the upstream sensors were powered directly via USB power banks, which were also used as ADC voltage references. While mostly stable, we observed some artifacts, which we put down to the upstream sensor’s supply voltage level shifting. This is noticeable in nine distinct jumps in the raw voltage signal of the PID sensors on the order of  −1 mV over the four days of experiments. Fortunately, we operated two redundant upstream sensors at all times. Since the observed  −1 mV are quite prominent over the noise floor, we were able to correct these anomalies. Our preprocessing script identifies the locations of these step-like shifts in the PID voltage signal on the upstream sensors and removes them by reattaching the right-hand side of the signal to the previous voltage level.

Since the PID-AH2 sensors from Alphasense require a static offset voltage compensation anyhow, the absolute voltage level is less critical. For the signal quality of the upstream sensors, which were later reused for background subtraction, we consider the correction as essential. The times and amplitudes of the nine steps are listed in Table 3.

**Table 3:** Upstream PID sensor voltage anomalies.

Date & Time	Amplitude [mV]	upstream idx
2023-03-08 09:35:03.343672	−0.750755	0
2023-03-08 09:11:39.251573	−0.804163	1
2023-03-08 10:16:37.815068	0.663778	1
2023-03-08 13:27:14.094516	−0.917081	0
2023-03-09 08:46:14.771962	−0.776696	0
2023-03-09 10:31:34.336694	−1.145970	0
2023-03-09 13:35:52.053562	−1.043733	0
2023-03-10 10:03:23.546736	−1.364177	0
2023-03-10 10:06:31.741394	−1.019318	0

Since the MOS sensors were measured as a voltage divider over the same voltage as the ADC’s reference, we would expect no change for moderate supply voltage changes. Users carefully observing the MOS sensor signal at the times listed in the table will, however, notice some glitches there, too. We put this down to the fact that while there should be no difference in reading the sensing resistance, we do expect a slight increase in heating power of the MOS integrated heating elements. The effect is minor and is hardly noticeable over the noise floor of the MOS sensors.

#### Step 2 – MOS: Voltage to Resistance

The MOS sensors’ sensing element resistances *R*_s_ change under the influence of gases. We measured the output voltage of a 5 V voltage divider consisting of said *R*_s_ and a load resistor *R*_load_ with our ADCs with a reference voltage of 5 V. Since we know the load resistances in our circuit, we were able to recover *R*_s_. For details, we refer to the source code and the schematics contained in the dataset.

#### Step 3 – MOS: Temperature and Humidity Compensation

MOS sensors are known to have severe cross-sensitivities to humidity and temperature (T&H). Temperature, humidity, and gas concentration have a complicated interplay in their effect on the sensing element resistance *R*_s_. From resistance alone, it is not possible to accurately compute the measured gas concentration. Numerous works on the T&H compensation for MOS sensors have been published, both in scientific literature^20–23^ and by the manufacturer^24,25^.

For this compensation, we included Bosch BME680 sensors on each sensor layer and the upstream sensors. As we could not find a comprehensive, universal guide to T&H compensation in the literature, we present here our simplified compensation model.

Generally speaking, a *decrease* in the sensing element resistance *R*_s_ of a MOS sensor can be caused by three factors:

1. an increase in ambient temperature,
2. an increase in relative humidity, or
3. an increase in gas concentration.

As MOS sensors are semiconductors, we model their temperature characteristic as negative-temperature-coefficient (NTC) elements. This is in line with the manufacturer’s AppNotes^24,26^. NTC behavior is commonly modeled using the “B parameter equation”

2 $${R}_{{\rm{s}}}(T)={R}_{0}\,{{\rm{e}}}^{B\left(\frac{1}{T}-\frac{1}{{T}_{0}}\right)},$$

parameterized by some known (absolute) temperature, e.g. *T*_0_ = 25 ^°^C = 298.15 K, and the *R*_0_ measured at temperature *T*_0_. For our purposes, we can reformulate this into

3 $${R}_{{\rm{s}}}(T)={R}_{\infty }\,{{\rm{e}}}^{-\frac{B}{T}}.$$

This equation has only two parameters, the material constant *B* and some limit *R*_*∞*_. We extrapolated the constant *B* from the characteristic temperature-resistance curve published in the manufacturer’s AppNote^24^, yielding a value of *B* = 3073 K. Note: even though the curve is of a MiCS-5525 sensor, we assume the sensor chemistry to be similar to the MiCS-5524 and MiCS-6814 sensors we used^27^.

For the range of relative humidity (RH) observed in our experiments (*H* = 50…80%RH), according to the manufacturers AppNotes, the influence of relative humidity can be modeled as a linear decrease of  −0.5% resistance per % of RH. So, knowing the resistance *R*_s,50%RH_, our model predicts

4 $${R}_{{\rm{s}}}(H)={R}_{{\rm{s}},50 \% {\rm{RH}}}-0.005(H-50){R}_{{\rm{s}},50 \% {\rm{RH}}}.$$

With our model for temperature and humidity influence laid out, we could then proceed as follows, for each of the 18 MOS sensors and for each experiment:

1. We determined the time ${t}_{\max }$ of the highest measured resistance *R*_s_ for each sensor specimen. This usually occurred shortly after turning on the wind tunnel. We treated this as our clean-air reference. We captured a *R*_0_, *T*_0_ and *H*_0_ at ${t}_{\max }$.
2. From this data point, we calculated an ${R}_{0,2{5}^{\circ }{\rm{C}},50 \% {\rm{RH}}}$, i.e. the resistance we would measure in clean air at 25 ^°^C and 50 %RH. To do so, we first compensated for humidity using Eq. (4), by plugging in *H*_0_ and *R*_s_(*H*) and solving for *R*_s,50%RH_. Then we determined *R*_*∞*_ using Eq. (3) by plugging in *T*_0_, and *R*_s,50%RH_ for *R*_s_(*T*), and used the same formula to derive ${R}_{0,2{5}^{\circ }{\rm{C}},50 \% {\rm{RH}}}$ for *T* = 25 ^°^C. We stored this value, along with *R*_*∞*_.
3. With the derived parameters, it was then possible to calculate
  5 $${R}_{0}(T,H)={R}_{{\rm{s}}}(T,H| c=0\,\,{\rm{ppm}}),$$
  where *c* is the concentration, by applying first Eq. (3) and then Eq. (4). This yielded a timeseries *R*_0_(*t*) of how *R*_s_ would evolve, given *T*(*t*) and *H*(*t*), in clean air, considering only the humidity and temperature changes.
4. Lastly, this allowed us to compute *R*_s_(*t*)/*R*_0_(*t*). This quotient is required for the conversion into concentrations in the next step. It can be considered the output of the temperature and humidity compensation.

This procedure assumes some independence between the three factors: temperature, humidity and gas exposure. We assume that the temperature dependence is driven by the NTC behavior, while the dependence on relative humidity is a consequence of water adsorption on the sensing element, and we assume these to be independent of each other. The influence of humidity and gas concentration is presumably not independent, as they both influence the semiconductor’s conductivity. As we performed our transformations on *R*_0_, so in the clean-air case, we maintain that this is still fair.

This can only be viewed as a first-level approximation of the true interactions of *T*, *H*, and *c* on *R*_s_(*T*, *H*, *c*). The authors invite further studies and experimental evaluation on this matter. If other compensation methods are of interest, readers are free to implement and utilize them instead and apply them to our published raw data. The source code for our compensation scheme is fully published (cf. Sec. Code Availability). Since this preprocessing served solely as a means to obtain a ppm-proportional quantity for the purpose of background subtraction, we maintain that it is fully sufficient for our needs here.

#### Step 4 – MOS: *R*_*s*_/*R*_0_ to Concentrations

The response to MOS gas sensors is highly nonlinear. There are several differing models to describe the relation of *R*_*s*_ to changing gas concentrations *c*. Models often focus on modeling the asymptotes for different regimes, of which we identified three:

1. Generally, for an increase in concentration, the resistance *R*_*s*_ follows some power law, depending on the sensor chemistry, the chosen target gas and other parameters. This behavior is usually captured by a straight line asymptote of negative slope in a log-log domain of *R*_*s*_ and *c*^20,21^.
2. For concentrations *c* → *∞*, some models describe *R*_*s*_ asymptotically approaching some *R*_*∞*_. This is the lowest resistance achievable by a MOS specimen, under maximal gas exposure^21^.
3. For concentrations *c* → 0, some models foresee the resistance asymptotically approaching a constant *R*_0_. This is the highest resistance achievable by a MOS specimen, which occurs in the absence of any reagents^20^.

What these models commonly do is express *R*_*s*_ as a fraction of *R*_0_, as *R*_0_ varies wildly between MOS sensor specimens, and the presence of gas causes sensors to lose resistivity proportional to *R*_0_, as described by some power law:

6 $${R}_{s}={R}_{0}\,{\left(1+kc\right)}^{-\beta },$$

with concentration *c*, *R*_0_ as the clean-air resistance, *β* > 0 as the (not necessarily integer) power law exponent, and k as a constant of dimension ppm^−1^. Parameter *β* marks the slope in a log-log plot of the characteristic curve of the sensor.

In the datasheet of the MiCS sensors, the manufacturer supplies the characteristic curves for various target gases. The curves are, however, approximated as purely linear in the log-log domain, essentially simplifying Eq. (6) to

7 $${R}_{s}={R}_{0}\,{\left(kc\right)}^{-\beta },$$

which is a fair approximation for high values of *c*. Note that, importantly, by doing so, Eq. (7) omits any asymptotic behavior for *c* → 0, meaning that $\frac{{R}_{s}}{{R}_{0}}$ diverges for zero concentration – or conversely, that given $\frac{{R}_{s}}{{R}_{0}}$, the computed *c* will never read zero. Since this is the best information available to the authors, we used these curves to convert from the fraction $\frac{{R}_{s}}{{R}_{0}}$ to concentrations in ppm.

Consequently, we proceeded by taking the quotient *R*_*s*_(*t*)/*R*_0_(*t*) from the previous steps, and applying the characteristic curves from the datasheets^18,19^ to it to obtain *c*. As the manufacturer states, this fraction is a constant for a set concentration level, i.e., not changing over time^25^. Because of this, we can recover values in ppm-equivalents of any choice of target gas available in the datasheets. We chose the following curves from the families: for the reducing gas channel (MiCS-5524), we used the ethanol curve; for the oxidizing gas channel (MiCS-6814, NO2), we used the NO_2_ curve; and for the NH_3_ channel (MiCS-6814, NH3), we used the ethanol curve. We would like to mention that these curves are only an approximation, and concentration curves obtained through individual sensor calibration would have been desirable, but are not available for this dataset.

#### Step 5 – PID: Offset Voltage Subtraction

The PID sensor signals were much less demanding to preprocess. First, the sensor already provided an analog voltage output. According to the manufacturer’s datasheet and AppNotes^28,29^, the sensors exhibit an offset in their output voltage, in the range of 46 mV to 60 mV. We determined this offset for each of the 6 PID sensors and each experiment by determining the lowest recorded voltage during the experiment, and subtracted it from the timeseries.

#### Step 6 – PID: Sensitivity Scaling Correction

One essential requirement for computing the concentration using the PID output signal is knowing the sensitivity *s*. According to the manufacturer, this value is ideally to be obtained through frequent calibration. Alphasense states a minimum sensitivity of $25\,\frac{{\rm{mV}}}{{\rm{ppm}}}$ in the datasheet of the PID-AH2, with a typical range of $25\,\frac{{\rm{mV}}}{{\rm{ppm}}}$ to $75\,\frac{{\rm{mV}}}{{\rm{ppm}}}$ and an average sensitivity of $55\,\frac{{\rm{mV}}}{{\rm{ppm}}}$. As we in our setup had neither the means to calibrate the PID sensors against a known gas concentration, nor the assurance that the PID sensors would not prematurely age due to condensation of the fog fluid inside the sensor housing, we had no reliable number for this sensitivity. Instead, we had to assume the value $s=55\,\frac{{\rm{mV}}}{{\rm{ppm}}}$. While the results stay concentration-proportional even for wrong values for *s*, this becomes an issue when comparing values from different sensor platforms or when subtracting the background obtained with a different PID. To alleviate this limitation, we introduced a correction factor *k*_PID,*i*_ for each sensor:

8 $${U}_{{\rm{PID}},i}^{{\prime} }(t)={k}_{{\rm{PID}},i}\ {U}_{{\rm{PID}},i}(t),$$

with *U*_PID,*i*_(*t*) being the (offset-subtracted) output voltage of the PID sensor and ${U}_{{\rm{PID}},i}^{{\prime} }(t)$ as its corrected counterpart. As will become clear later, this is equivalent to scaling the sensitivity as

9 $${s}_{i}=\frac{1}{{k}_{{\rm{PID}},i}}\cdot 55\,\frac{{\rm{mV}}}{{\rm{ppm}}}.$$

We stored ${U}_{{\rm{PID}},i}^{{\prime} }(t)$ in the dataset as voltage_scaled.

The factor *k*_PID,*i*_ was determined as follows. In the recorded voltage timeseries, we looked for the first sharp increase. This occurs at the time when the sensor probe first crosses in front (downwind) of the gas source. We denote the time just before reaching this spike as *t*_1_. For the sampling experiments, this took on the order of 20 min, as there were some Y-Z rows to scan upstream of the source. During this time, we saw a meaningful increase in background concentration in the tunnel – importantly, without any particular sensor experiencing any direct exposure. This is due to the tunnel being a closed circuit, with the source at this point still directly downstream of the sensors. When we compare all PID sensors, their concentration readings should all rise to the same level, as they all experience the same background concentration. Since we saw that this is not the case, we set *k*_PID,*i*_ such that

10 $${k}_{{\rm{PID}},i}=\frac{\frac{1}{6}{\sum }_{i=1}^{6}{U}_{{\rm{PID}},i}({t}_{1})}{{U}_{{\rm{PID}},i}({t}_{1})}.$$

This had two effects: First, it ensured that all PID sensors follow roughly the same slope during the first minutes of the experiments, until *t*_1_. This also ensured similar overall slopes over the course of the 2 h of the sampling experiments. Secondly, it took advantage of the fact that by averaging over all 6 sensors, we would expect to get close to the population average of $55\,\frac{{\rm{mV}}}{{\rm{ppm}}}$ stated by the manufacturer.

This method was only feasible for the “sampling experiments”. For all other recordings, we kept it fixed at *k*_PID,*i*_ = 1. Also note that we performed this method on a per-experiment basis. Users interested in correlating the correction factors across experiments are encouraged to do so, keeping in mind that PID sensors were swapped according to Table 2. Another option is to look at the spatial grid cells visited by PID sensors of two different layers throughout the experiment, which were strategically added every second Y-Z sampling row (see Sec. Sampling Patterns).

#### Step 7 – PID: Voltage to Concentration

Due to the linear sensor characteristics of PID sensors, the resulting voltage could then be multiplied by a sensitivity to obtain a concentration:

11 $$c({U}_{{\rm{PID}}})=\frac{{U}_{{\rm{PID}}}}{s}\,{\rm{RF}},$$

where *U*_PID_ is the (offset-subtracted) PID voltage, *s* is the sensitivity of the PID, usually given in $\frac{{\rm{mV}}}{{\rm{ppm}}}$, and RF is some response factor specific to the gas. These can be looked up in a manufacturer specification table^30^, which states a RF_Ethanol_ = 3 and RF_PG_ = 11. The same document also provides a formula for gas mixtures, which allowed us to compute the RF of the mixture present in our fog fluid.

At this point, we would like to mention that the values in the table did not match our empirical observations fully. While both constituents, PG and Ethanol, excited some response in the PID sensor, the sensitivity to pure Ethanol was much stronger than that of pure PG – in contradiction to the ratio stated in the manufacturer’s table. Whether this was due to aerosolization instead of vaporization, an effect of the filter membrane, or other effects, is unknown to the authors. The published dataset contains concentration equivalents for the pure-ethanol, pure-PG, and the mixture case. They differ only in a proportionality factor.

#### Step 8 – Background Concentration Subtraction

We placed two upstream sensors in the foreland of the wind tunnel (cf Fig. 7). These provided two redundant baseline readings of the concentrations (and PHT values) in the air in the tunnel circuit. This is important because we accumulated some background concentration during the duration of each experiment. Using these upstream sensors, we could subtract this. This subtraction should be performed on the concentration level, i.e., in ppm-proportional units. To remove the background concentration in our preprocessing, we combined the two upstream sensors into one composite timeseries of the (T&H-compensated) background concentrations by computing the average between them. Then we filtered it with a rolling average filter (window size: 30 s) to suppress the influence of noise. As the background is a very slowly changing signal, this should introduce no adverse effects. This signal was then subtracted from the concentration signals from the previous steps.

This signal represents the increase in concentration caused by the influence of the gas plume emanating from the gas source. Values above zero indicate a local increase in concentration. As this is a *relative* signal, it is also possible to observe negative values. These would indicate a drop below the background level in concentration. We did observe this in some of the timeseries, primarily in two cases: For some experiments, the concentration of PID and MOS dropped below baseline levels for layers 2 and 3, which were the highest and experienced little to no direct exposure to the plume. Whether this effect was due to inaccuracy of the baseline compensation, or an actual physical phenomenon, i.e., due to the vertical wind speed profile or due to gravitational/buoyant separation, is suggested as a topic for future inquiry. The second effect is visible in the signal of the NO_2_ channel of the MiCS-6814 sensor. As this sensor is sensitive to oxidizing gases, and the fog we released was primarily reducing, the plume acted as an “anti-oxidizing” gas, thus explaining the negative relative concentration values.

Readers interested in background-subtracted PHT readings would also be able to compute those; here, we have incorporated the T&H readings directly into our concentration computation instead.

#### Step 9 – Voxelization of Time Series Data

During recording, whenever the probe arrives at a measuring position, the machine motion buffer was flushed, ensuring the machine’s position matches the expected state exactly. The data was then tagged from this moment onward. When the designated sampling time had elapsed, tagging ceased and motion was resumed. The tagset comprises:

- x the current *x*^G^ of the sensor platform’s center point,
- y the current *y*^G^ of the sensor platform’s center point,
- z the current *z*^G^ of the sensor platform’s center point,
- module a string indicating which sensor platform this sensor is mounted to.

Using tag module, data was separated into the 4 CSV files/array elements we will introduce in Sec. Data Records. Using this tagset, it is easy to extract the timeseries recorded by one sensor in one cell. For most grid-based sampling patterns, these timeseries constitute 5 s of measurements, i.e., 50 samples. To obtain a 3D gridmap of concentrations (& temperatures, humidities, pressures, wind vectors), the most basic approach is to compute the arithmetic mean of all samples within one of these sub-timeseries. Doing so yielded one scalar per measured quantity and grid cell, or one 3D vector in the case of the wind velocity. Each quantity can thus be expressed as a voxelized volumetric image. These “voxel maps” were exported as part of the dataset.

### Suggested Further Processing

The preprocessing steps presented in this section prepare the dataset for most applications. Concentration timeseries as well as spatial snapshots of the gas dispersion process can be analyzed. Further auxiliary data are available, like spatially resolved pressure, humidity and temperature measurements. A full spatial map of 3D wind vectors constitutes a flow field reconstruction around the model landscapes. All experiments are thoroughly augmented with metadata.

For some advanced use cases, some additional processing steps can be implemented. We will briefly discuss these, providing additional insights where necessary.

#### Layer Cross-Calibration, Offset Compensation, Sensor Baseline Compensation

Users interested in studying the effects of inter-sensor variances have the option to do so using this dataset. Since we used four sensor platforms simultaneously, each exhibiting individual baseline resistances *R*_0_ for the case of the MOS sensors, or sensitivity factors *s* for the PID sensors, the dataset offers many opportunities to study drift, aging and inter-sensor variability.

We included means to cross-check pairs of sensor layers against each other in the same physical location in our sampling patterns. To this end, we let pairs of sensors overlap at the end of every second Y-Z row of the grid-based sampling patterns (cf. Sec. Sampling Patterns).

Additionally, we recorded the response of the sensors to clean-air exposure (“purging runs”). This may allow users to validate the clean-air *R*_0_(*t*) signals of all MOS sensors.

#### Wind Sensor Cross-Calibration

Our setup includes 4 ultrasonic anemometers and 3 pitot tubes. At the beginning of all sampling experiments, the sensor probe was positioned as close as the machine travel allowed to the location of the pitot tubes A and B. This enables users to cross-validate the ultrasonic anemometers among each other, as well as against the pitot tube readings. Note that pitot tube C was responsible for controlling the wind speed in the tunnel.

#### Impulse Response Compensation

The time that sensors remain in each cell was short in relation to the response/recovery time of the sensors, i.e., the characteristic time of the sensor’s step responses. Some trail-off effects after strong exposures have been captured as part of the dataset. If one is able to cancel out or reduce these effects, this can greatly aid fast-moving olfactory robots. One approach is to deconvolve the sensor signal with its impulse response, as put forward in^16^. We encourage further studies in this vein, using this dataset.

#### Timing Alignment

Users who require ultra-precise timing information of when sensor signals were acquired may further process the timestamps attached to the data records. The sensor layers derived their timing signal from the single-board computer that is running the database, CNC controller, and sampling software. There, a Python script generated the 10 Hz clock, using a BackgroundScheduler from the apscheduler package. The sensor layers were internally synchronized: The measurement acquisition was triggered by an RS-485 broadcast. It can thus be assumed that acquisitions were initiated synchronously to the *μ*s at least. Capture of all channels was then completed in sequence within approx. 2 ms by each of the multiplexing ADCs. The database records were tagged with the NTP time at which the trigger signal was sent out by the single-board computer. Since each measurement was also tagged using the trig tag, a monotonously increasing counter of trigger signals, users may be able to infer even more precise timing information. Whether individual acquisitions were lost due to signal interferences on the RS-485 bus, can also be detected and compensated using this tag.

The upstream sensors were synchronized to the same NTP server. Their clock was generated in Python, using time.sleep() to pad the loop time after measurement acquisition to 100 ms. Data was sent to the database over the air and tagged with the NTP time of the upstream module.

## Data Records

The full dataset is provided in our Zenodo repository^31^. At the root of the repository, we provide a document named csv_header_specification.pdf with details on the dataset structure, starting with a table of the 39 different experimental runs we conducted. This list includes a unique chronological index for each run, start time and duration, a type, wind speed, sampling time, setup name, and a directory path. The type is one of *Sampling Experiment / Fly-Through Experiment / Purging Run*, indicating what kind of recording has been performed in this run (see Sec, Sampling Patterns). The setup name corresponds to the setups outlined in Sec. Sampling Patterns. The specified directory then contains the respective dataset. This table is also provided in machine-readable form in overview_table.csv.

**Fig. 12:**
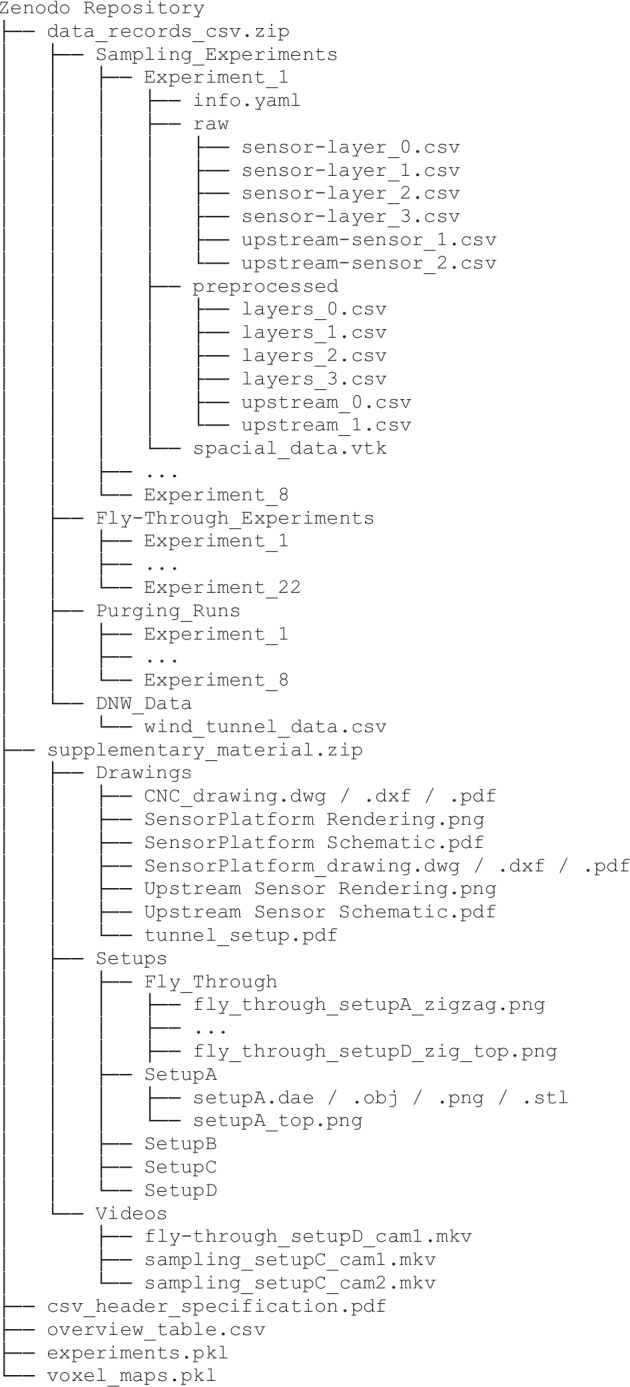
Repository Structure.

Figure 12 provides an outline of the structure of the repository. The primary experimental data is contained in the three directories Sampling_Experiments, Fly-Through_Experiments, Purging_Runs, named after the experiment type. The experiments are numbered sequentially; refer to the overview table. Each subdirectory Experiments_N contains an info.yaml, containing some experiment metadata, as well as a raw and preprocessed directory. These contain individual .csv files for each sensor layer or upstream sensor, respectively.

The raw data .csv files contain timeseries of the recorded voltages. In the processed data .csv files, each sensor timeseries is split up into several columns, containing both the raw voltage, as well as all derived quantities up to the concentration signal in ppm and the background-subtracted ppm signal. The four types of .csv files (layer/upstream, raw/preprocessed) contain a set of column headers, which are enumerated and annotated in the csv_header_specification.pdf of the dataset for reference. An example of the contained timeseries is shown in Fig. 15. If users require partial preprocessing, or any other modification or extension in the preprocessing chain, they may use the scripts we make available and introduce in Sec. Code Availability.

From the preprocessed timeseries, we can generate a voxelized gridmap of the 3D environment, where we aggregate and average all values that have been recorded in a single grid cell. These voxel maps are provided in VTK format as voxel_map.vtk, where the position of each cell is defined by the X/Y/Z-indices. When opening these VTK files in an application like ParaView, users can select any of the computed quantities per grid cell, which are contained in “Data Arrays” and are analogously named to the .csv headers, e.g., MiCS5524_ppm (scalar), PID-sensor_voltage (scalar), wind (3D vector), etc. If users are interested in anything but the averaged readings in the cell, i.e., the standard deviations, the entire sub-timeseries, etc., they may modify and use the scripts we used to generate the voxel maps, according to our examples. These scripts can again be found in Sec. Code Availability. This voxelization is only possible in those experiments that actually perform a grid-based sampling pattern. For the fly-through experiments, which do not visit regular grid cells, and the purging runs, only the timeseries are provided.

**Fig. 13:**
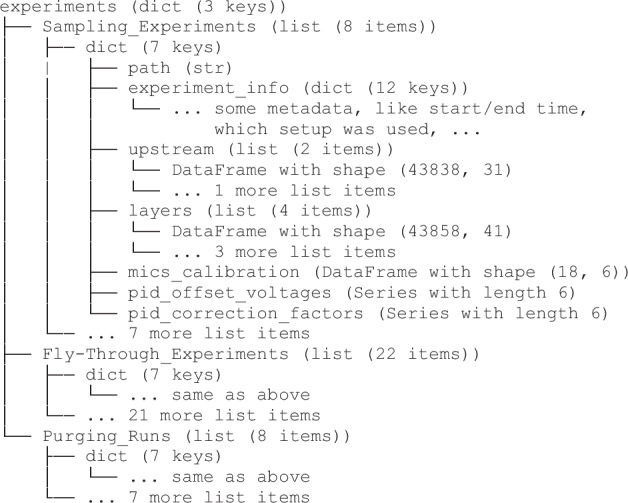
Structure of the experiments data structure contained in experiments.pkl.

**Fig. 14:**
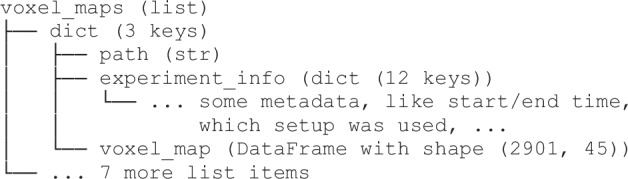
Structure of the voxel maps data structure contained in voxel_maps.pkl.

For the convenience of users working with the dataset in the programming language Python, we also provide the full set of raw and preprocessed timeseries as a Python “pickle” file, experiments.pkl, at the root of the repository. In there, we make use of data frames as part of the Python library pandas. The structure of this data is laid out in Fig. 13. In the same format, users will find a voxel_maps.pkl file containing the rasterized version of the data. The data structure is summarized in Fig. 14.

Furthermore, the directory DNW_Data contains a .csv file containing the data provided by the wind tunnel operator DNW. For an overview of the contained columns, refer to csv_header_specification.pdf. In the directory Drawings, users will find technical drawings and schematics in various formats for the wind tunnel setup, the CNC sampling machine, and the sensor circuit boards. The directory Setups contains 3D model files for the scale model landscapes used in setups A through D, image renderings of them, as well as image renderings of the fly-through flight paths. Under Videos, we supply some time-lapse videos as examples of how experiments were performed.

## Technical Validation

The presented dataset consists of 39 experiments in total: 8 rasterized sampling experiments, 22 fly-through experiments, and 9 purging runs. For each of these experiments, 6 groups of timeseries have been recorded for the different sensor PCBs: 4 sensor layers plus 2 upstream sensors. Each of those consists of 4 gas sensor columns, 3 columns for the PHT readings, plus, in the case of the sensor layers, 6 further columns for the 3-dimensional wind vector and another set of PHT readings output by the anemometer. Additionally, for each experiment and sensor type, there are 4 (for the MOS sensors) or 5 (for the PID sensor) derived timeseries from the preprocessing steps.

### Manual Inspection

**Fig. 15:**
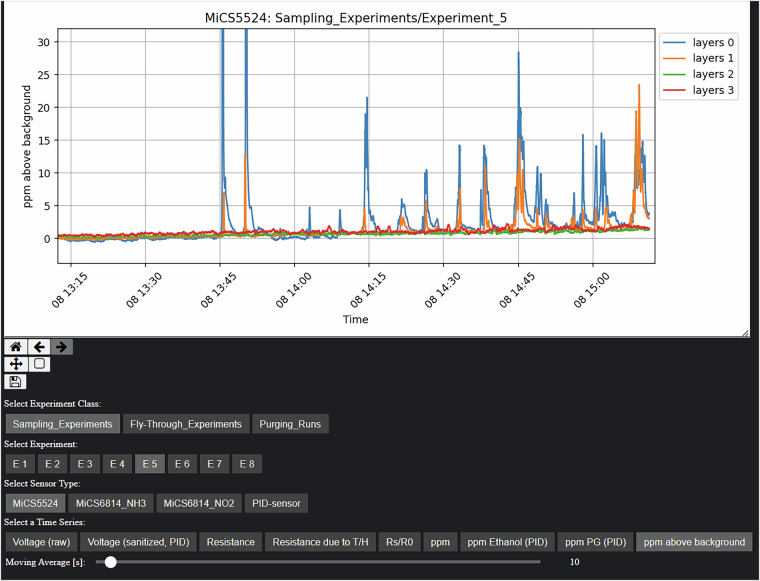
Screenshot of the Jupyter notebook for inspecting the different timeseries.

To manually inspect and validate these timeseries, we prepared an interactive Jupyter notebook, provided as part of the source code repository (cf. Sec. Code Availability). This notebook allows users to display the timeseries by selecting any of the 39 experiments, the sensor type, and the desired timeseries column. A slider widget lets users apply some moving average filtering, with which low-frequency trends and high-frequency sensor responses can be separated. For illustration purposes, a screenshot of this notebook is shown in Fig. 15.

Inspection shows that the timeseries are complete and free of outliers for all experiments. Responses for the PID sensor are found to peak as high as 40 ppm above background, with most readings, however, remaining below the 5 ppm mark. We deem the absolute ppm values of the MOS sensors to be less trustworthy. Nonetheless, their signals show a similar trend. There are significant, prominent spikes visible when the probe traverses the gas plume, and different levels of intensity are discernible. As it is one of the main purposes of this dataset to allow for the study of these low-cost, off-the-shelf sensor units in comparison with the “gold standard” of the PID sensor, this highlights the value of this dataset. It can aid research that would allow MOS sensors to be used in their stead.

Also visible is the slow recovery of the MOS sensors after being exposed to high gas concentrations. Their values take a long time after leaving grid cells inside the plume to drop back to their background level. This highlights the need for impulse response compensation as alluded to in Sec. Impulse Response Compensation. A similar effect is, for some experiments, visible in some of the PID sensors – especially so for the ones on the lower two sensor platforms. The physical mechanism for this will be different, though; we suspect condensation of the fog fluid inside the sensors’ housings, which is also what prompted us to replace PID sensors (cf. Table 2, Sec. Sensors and Sensor Layers).

**Fig. 16:**
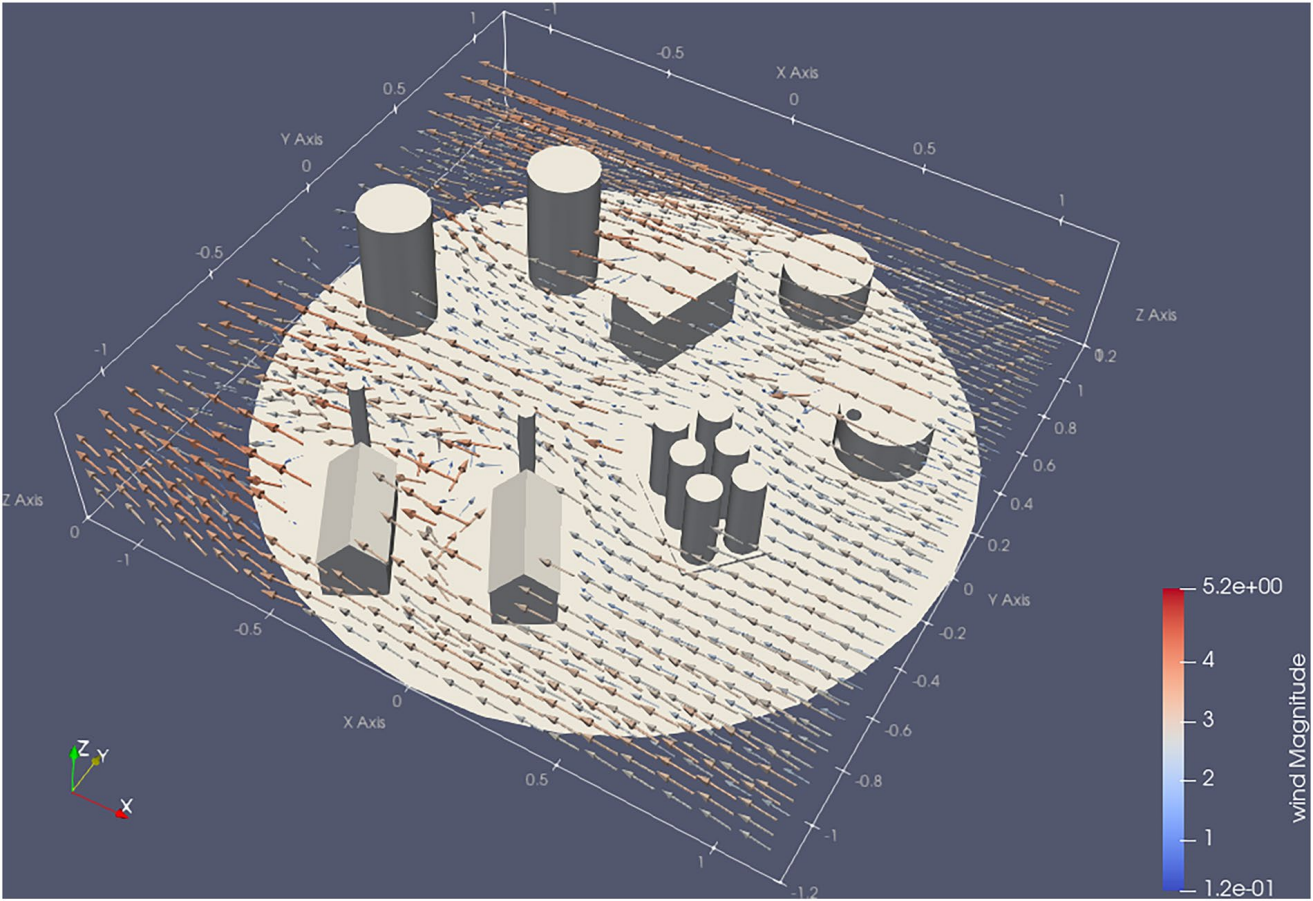
Screenshot of air flow measurements in ParaView (only the measurements below *z*^*G*^ = 0.3 m are shown). In addition, the STL model of setup D as contained in the dataset is depicted.

**Fig. 17:**
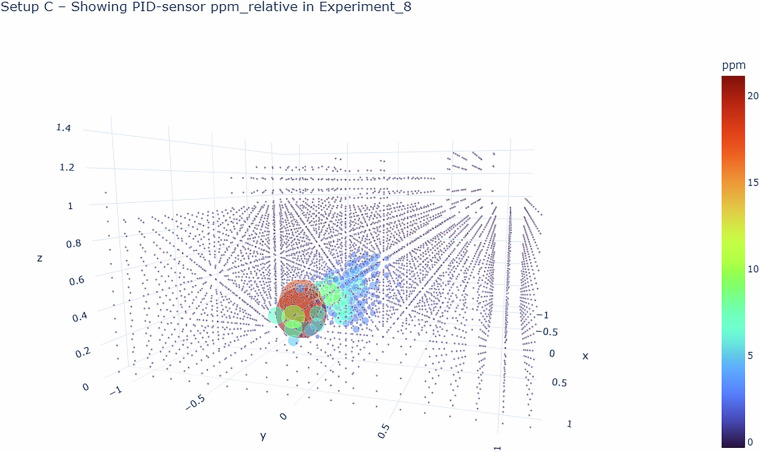
3D rendering out of the Jupyter notebook for inspecting the 3D reconstructions of the wind tunnel volume.

A second way to manually inspect the validity of the data records is to load the generated voxel maps into a 3D visualization software like ParaView (cf. Fig. 16), or by inspecting the voxel maps in the Jupyter notebooks supplied in our source code repository. In the visualization, the plume structure is clearly visible in the 3D reconstructions (cf. Fig. 17). It shares similarities with the visual feedback that we get from the fog; see the photo shown at the start of this article (cf. Fig. 1).

When inspecting the voxel map, small differences in cells recorded by different sensor layers may become visible; for example, see sampling experiment 1, the PID sensor signal at the boundary of the test section. Through our background concentration removal during preprocessing, this effect is minimized. Users of the dataset may study methods of reducing these cross-sensor differences further, or even attempt calibration transfer methods between them^32^.

### Validation of Sampling Periods

**Fig. 18:**
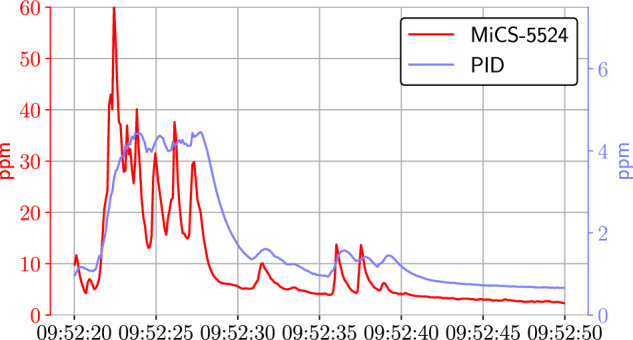
PID vs. MOS dynamic response to a gas stimulus. The sensor platform is entering the gas plume. The resulting concentration signals are shown here.

MOS sensors are known for their slow response and recovery behavior, while PIDs can be considered fast sensors^33–35^. However, as explained above, constrained by the overall experiment time and our goal to achieve a high resolution with our sampling grid, the time per grid cell had to be limited to an acceptable minimum. In general, robotic gas sensing applications also face the challenge of short sampling times. For the complex setups C and D, we decided on 5 s sampling time per cell. This raises the question of whether this time is sufficient for the MOS sensors to capture the gas concentration levels at the measured locations. The collected data, namely the comparison between MOS and the “gold standard” PID, shows that indeed the MOS Sensors can capture dynamic changes in the concentration levels below 5 s. As an example, Fig. 18 shows the sensor signals for the MiCS-5524 and the PID sensor (Layer 1) when passing through the plume behind an obstacle. As can be seen, the MOS sensor can clearly follow the concentration changes that are detected by the PID sensor. So, especially for response times, we can consider the 5 s as enough. However, please note that the dataset also contains experiments with up to 30 s sampling time per cell. This data can be the foundation for future work on estimating minimal sampling times for robotic sensing applications.

## Data Availability

The Red:Vapor dataset is published and available for download on Zenodo^31^.

## Code availability

The source code that has been used in this work for data processing and analysis is available as part of our GitHub repository at https://github.com/DLR-KN/red-vapor, along with examples, a quick-start guide and usage instructions. In reference to this article, the preprocessing steps described in Sec. Preprocessing can be found in the preprocessing Python scripts. This repository also contains Jupyter notebooks for visualizing the raw and preprocessed data as timeseries as well as 3D voxelizations, which can be explored interactively online. The description in the README may serve as a starting point of how to use the dataset and the source code repository.
